# A Multiplex Molecular Cell-Based Sensor to Detect Ligands of PPARs: An Optimized Tool for Drug Discovery in Cyanobacteria

**DOI:** 10.3390/s23031338

**Published:** 2023-01-25

**Authors:** Inês Páscoa, Rita Biltes, João Sousa, Marco Aurélio Correia Preto, Vitor Vasconcelos, Luís Filipe Castro, Raquel Ruivo, Isabel Cunha

**Affiliations:** 1 CIIMAR/CIMAR-Interdisciplinary Centre of Marine and Environmental Research, University of Porto, 4450-208 Matosinhos, Portugal; 2 ICBAS-Instituto de Ciências Biomédicas Abel Salazar, University of Porto, 4050-313 Porto, Portugal; 3 FCUP-Faculty of Sciences, Department of Biology, University of Porto, 4169-007 Porto, Portugal

**Keywords:** luciferase, reporter gene, PPAR agonist, bioactive compound, assay-guided drug discovery, limit of detection, EC50, screening, dual agonist, biosensor

## Abstract

Cyanobacteria produce a wealth of secondary metabolites. Since these organisms attach fatty acids into molecules in unprecedented ways, cyanobacteria can serve as a novel source for bioactive compounds acting as ligands for Peroxisome Proliferator-Activated Receptors (PPAR). PPARs (PPARα, PPARβ/δ and PPARγ) are ligand-activated nuclear receptors, involved in the regulation of various metabolic and cellular processes, thus serving as potential drug targets for a variety of pathologies. Yet, given that PPARs’ agonists can have pan-, dual- or isoform-specific action, some controversy has been raised over currently approved drugs and their side effects, highlighting the need for novel molecules. Here, we expand and validate a cell-based PPAR transactivation activity biosensor, and test it in a screening campaign to guide drug discovery. Biosensor upgrades included the use of different reporter genes to increase signal intensity and stability, a different promoter to modulate reporter gene expression, and multiplexing to improve efficiency. Sensor’s limit of detection (LOD) ranged from 0.36–0.89 nM in uniplex and 0.89–1.35 nM in multiplex mode. In triplex mode, the sensor’s feature screening, a total of 848 fractions of 96 cyanobacteria extracts were screened. Hits were confirmed in multiplex mode and in uniplex mode, yielding one strain detected to have action on PPARα and three strains to have dual action on PPARα and -β.

## 1. Introduction

Peroxisome proliferator-activated receptors (PPARs) are nuclear receptors (NRs), with ligand-inducible transcription factor function, that control the expression of target genes [1,2,3]. PPAR target genes are generally associated with, but not limited to, lipid and carbohydrate metabolism (for detailed reviews see references [2,3]). As such, these pleiotropic receptors have been implicated in the regulation of several physiological and metabolic processes: including, energy homeostasis, cellular proliferation and differentiation, development and inflammation, among others [3]. These NRs heterodimerize with retinoic X receptors (RXRs) and, upon ligand binding, modulate the expression of downstream target genes, contingent on the presence of co-repressors or co-activators [4]. PPARs present a highly conserved DNA binding domain (DBD) that binds to specific sequences of DNA upstream of target genes, known as response elements, and a conserved ligand binding domain (LBD) involved in the dimerization of the receptor, and in the transduction of a ligand signal into the transcription of the target genes (i.e., transactivation) [5,6]. In humans, three PPARs paralogous genes have been identified (PPARα, PPARβ/δ and PPARγ) with different tissue expression patterns, ligands affinity, and target genes [7].

During the last years, PPARs have received broad interest from the pharmaceutical industry, mainly for the treatment of different metabolic pathologies [8]: such as type 2 diabetes, dyslipidemia, hypercholesterolemia, cardiovascular metabolic diseases, atherogenic and inflammation of vascular wall and immune cells [8,9]. Additionally, PPARs have also been linked to tumorigenesis and oncogenesis regulation [10], and neurological diseases [11,12]. Since the LBDs of the three isoforms are highly similar, ligands can be pan-, dual- or isoform-specific [13,14]. Thus, the collateral effects observed over some PPAR agonists used as drugs, such as those of the thiazolidinedione and fibrate families, urge demand for new PPAR drugs [15].

Endogenous ligands of PPARs are lipids or lipid derivatives, including phospholipids, endocannabinoid-like molecules, lipoprotein products, fatty acids (FAs), polyunsaturated FAs, and oxylipins; the latter resulting from FAs metabolization by cyclooxygenase, lipoxygenase and CYP450 enzymes [14,16,17,18]. Cyanobacteria produce a wealth of secondary metabolites with high biotechnological relevance, including pharmaceuticals, having been pointed out in the last decade as one of the most promising groups of microorganisms in this regard [19,20,21,22,23,24]. Among them, FAs are metabolized by these prokaryotic organisms in unusual ways, revealing unprecedented FA-incorporation in various molecules [25,26,27] that become unique and may simultaneously cause novel modulatory effects in one or various PPARs [28,29].

The present work intends to expand and validate a cell-based transactivation sensor of NR’s activity [30,31,32,33] increasing its throughput and sensitivity, to be used as a screening tool for natural compounds from biological samples, in drug discovery campaigns, namely, to detect new PPARs ligands. Generally, these genetically encoded luminescent sensors use a pair of vectors that are transfected into cells, in this case COS-1 cells: a first vector producing the fusion protein with the NR’s LBD and a yeast galactosidase-responsive transcription factor (GAL4) DBD (GAL4-DBD/NR-LBD), co-expressed with a constitutive reporter gene serving as an internal control for transfection efficiency, and a second vector including a upstream activation sequences (UAS), recognized by the GAL4-DBD, which controls the expression of a second reporter gene. In reporter gene assays, luciferases are frequently selected as reporters because of their ease of use, low endogenous activity, and wide dynamic range [34,35]. Recently, new luciferase reporter enzymes have been found [36,37] and those already in use have been engineered to improve performance and signal stability—two critical features of these systems.

**Figure 1:**
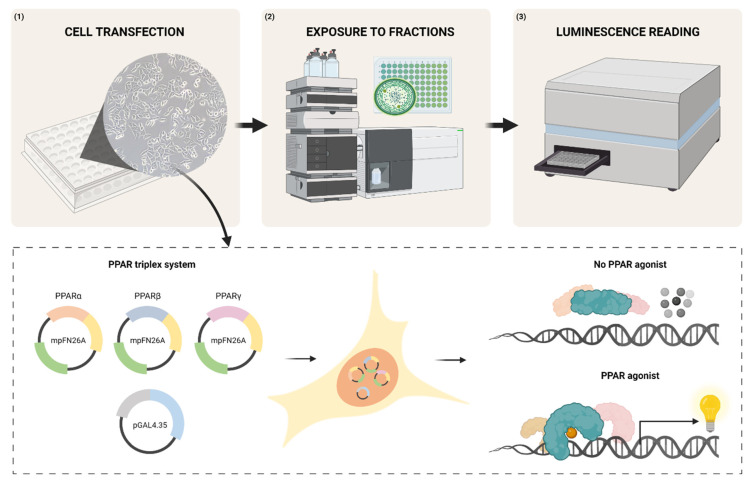
Schematic description of the transactivation assays methodology. (1) COS-1 cells are co-transfected in 96-well plates, with the vectors of the sensor system to be used, using Lipofectamine^®^ 2000 (Invitrogen™); (2) After 5 h, COS-1 cells are exposed for 24 h either to solvent control, to the reference agonists of each PPAR, or to the samples to be tested; (3) The luminescent activity of each vector’s luciferase is quantified, using the respective luciferase reporter kit in a microplate reader.

In the present work, an optimized version of a well-established transactivation assay protocol [30,38,39,40] was used to detect PPARs’ agonist activity, after validation with reference ligands of each of the three PPARs, and used to screen extracts of Cyanobacteria strains for the presence of bioactive compounds with such activity (Figure 1). The innovations developed are (1) technological, due to the modifications made on the vectors of the reporter system, namely changing the luciferases and the promoters to obtain a more stable and sensitive reporter system and are also (2) methodological, given the simultaneous transfection of COS-1 cells with vectors that transcribe for the 3 PPARs, which allows for the identification of agonist of the 3 PPAR in parallel (multiplexing), making it more efficient and suitable for use in screening assays to guide drug discovery (Figure 1). Particularly in the case of PPARs, for which inter-receptor ligand binding domains (LBDs) are highly similar, ligands can be pan-, dual- or isoform-specific. In the two former cases, the signal obtained by the presented sensor system results from the cumulative activity of 2 or 3 receptors, giving rise to a stronger signal when synergy occurs. There was also a reduction of the assay volume, and consequent cut on reagents use. Screening assays for drug discovery are very time consuming and expensive. Increasing assays’ throughput helps in reducing costs and increasing the amount of information obtained by unit of effort. The developed sensor was then validated with specific PPAR agonists (GW7647, GW501516, and rosiglitazone) and the Z′-factor used on data analysis.

Afterwards, the sensor was tested by assessing PPARs’ modulation by cyanobacteria fraction—96 cyanobacteria strains yielding 768 fractions plus 80 fractions of 10 Nostocales strains that were simultaneously cultivated in Z8 medium with and without nitrogen supplementation, making a total of 848 fractions, from the Cyanobacteria Natural Products Library (LEGE-NPL) [41], having originated from strains of the Blue Biotechnology and Ecotoxicology Culture Collection (LEGE-CC) [22]. Primary hits, obtained in multiplex mode, were confirmed in a second assay, also in multiplex mode, resulting in the identification of six fractions of five cyanobacteria strains with PPARs’ transactivation activity: *Sphaerospermopsis* sp. LEGE 02266, cf. *Oxynema acuminatum* LEGE 06078, *Cyanobium* sp. LEGE 06139, *Oculatella* sp. LEGE 06141, and *Nodosilinea* (*Leptolyngbya*) *antarctica* LEGE 13457. These strains were then assayed in uniplex mode to validate the modulation of each individual PPAR when exposed to the compounds present in each fraction. One strain displayed action on PPARα, and three strains had dual action on PPARα and -β. Uniplex mode refers to when the activity of only one PPAR is being measured, while multiplex mode refers to when multiple PPARs are being measured simultaneously. It depends on the presence of experimental vectors containing the Ligand Binding Domine of only one PPAR, or equal parts of various PPARs’ LBDs, in the present case PPARs α, β and γ, simultaneously, one third of each.

In the future, these fractions will be alternately subjected to subfractionation and scrutinized using the sensor here developed, to guide drug discovery assays in the isolation and identification of the compounds responsible for the observed PPARs transactivation hits. In conclusion, the proposed methodology is more efficient, sensitive and specific in identifying PPAR agonists, and it is suitable for use in screening assays to guide drug discovery.

## 2. Materials and Methods

### 2.1. Construction of Plasmid Vectors

Two cell sensor systems were used in the course of this work, using vectors acquired from Promega. One of the systems makes use of pBIND (AF264722) and pGL4.35 (GQ229577) vectors, already in use in our laboratory to quantify PPARs activity in cells, serving as comparative system for the new sensor to be developed. The new sensor system used the plasmids pFN26A (pBIND) hRluc-neoFlexi^®^ (GQ229578), pGL4.54[luc2\TK] (KM359769), pGL4.35 (GQ229577) and pNL1.2[NlucP] (JQ437371), as template parts to construct two new plasmids: the mpFN26A[Fluc] and mpGL4.35[Nluc]. This procedure was accomplished by NZYTech according to our instructions (Figure S1 of Supplementary Materials). Fluc stands for firefly luciferase, Nluc for Nanoluc^®^ luciferase and RLuc for *Renilla* luciferase.

### 2.2. Isolation and Cloning of PPARα, -β and -γ

The hinge and LBD regions of *Homo sapiens* PPARα, -β and -γ (XM_011530239.2, NM_006238.5 and NM_138712.4, respectively) genes were amplified by PCR, using Phusion High-Fidelity DNA Polymerase (2 U/µL; Thermo Scientific™, Waltham, MA, USA) and specific primer pairs, containing restriction sites for specific enzymes (Table S1). The PCR reaction comprised an initial step at 98 °C for 30 s, followed by 30 cycles at 98 °C for 5 s, 60 °C for 10 s and 72 °C for 15 s, ending with an extra step at 72 °C for 5 min.

The amplified products and the pBIND and mpFN26A[Fluc] vectors were double digested with the respective restriction enzymes, followed by 30 min at 80 °C to inactivate the enzymes. After isolation and digestion, hinge and LBD regions of PPARα, -β and -γ were inserted individually into pBind[Rluc] and mpFN26A[Fluc] vectors, using T4 Ligase (Promega, Madison, WI, USA), incubated at 4 °C, and then cloned in NZY5α competent cells (NZYTech, Lisboa, Portugal). A colony screening was performed to determine which colonies had the correct insertions, and the DNA sequence on positive colonies was confirmed by Sanger sequencing (Eurofins Genomics, Ebersberg, Germany).

### 2.3. Transactivation Assays

COS-1 cells were maintained in DMEM with phenol red (PAN-Biotech, Aidenbach, Germany) supplemented with 10% fetal bovine serum (PAN^-^Biotech), and 1% penicillin and streptomycin (PAN^-^Biotech) in a humidified atmosphere at 37 °C and 5% CO_2_.

All transactivation assays performed during the present work followed the same methodology (Figure 1). On the day before transfection, COS-1 cells were seeded in 96-well plates at a density of 4 × 10^4^ live cells per well, in supplemented DMEM with phenol red. Past 24 h, COS-1 cells were co-transfected with the vectors of the sensor system to be used, either the original system (pBIND[Rluc]/pGL4.35[Fluc]) or the new sensor system (mpFN26A[Fluc]/mpGL4.35[Nluc]), using Lipofectamine^®^ 2000 (Invitrogen™, Waltham, MA, USA), in Opti-MEM reduced serum medium (PAN^-^Biotech). After 5 h of transfection, COS-1 cells were exposed either to dimethyl sulfoxide (DMSO, solvent control; concentration never exceeding 0.5% in the well plate), to the reference agonists of each PPAR, or to the samples to be tested, both dissolved in DMSO, in DMEM without phenol red (PAN^-^Biotech) supplemented with 10% fetal bovine serum charcoal-treated (PAN^-^Biotech) and, 1% penicillin and streptomycin (PAN^-^Biotech). After 24 h of exposure, the luminescent activity of the luciferases of each vector of the system were quantified using the respective luciferase reporter kit and a microplate reader (Synergy HT Multi-Mode; BioTek, Winooski, VT, USA).

#### 2.3.1. Optimization of Vectors Ratio in mpFN26A[Fluc]/mpGL4.35[Nluc] System

Firefly (Fluc) and Nanoluc^®^ (Nluc) luciferases were used in the novel mpFN26A[Fluc]/mpGL4.35[Nluc] sensor system, in replacement for *Renilla* luciferase (Rluc) and Fluc, used in the initial pBIND/pGL4.35[Fluc] system, in an attempt to achieve a better performance upon adequate optimization. In this new sensor system, vectors pBIND[Rluc] and pGL4.35[Fluc] were replaced by the modified vectors mpFN26A[Fluc] and mpGL4.35[Nluc], respectively.

An assay was performed to optimize the vector co-transfection ratio, using 2:1, 1:1, 1:4 and 1:8 ratios between mpFN26A[Fluc/PPARγ] and mpGL4.35[Nluc]. This optimization was only performed with PPARγ, chosen randomly, having assumed that the results would be similar regardless of the PPAR used. The quantity of mpGL4.35[Nluc] (20 ng per well) was constant in all wells, while the amount of mpFN26A[Fluc] varied from 40 ng/well in the 2:1 ratio, to 2.5 ng/well in the 1:8 ratio. A carrier vector (pUC.19; L09137; Thermo Scientific™) was also co-transfected, in adequate quantities, to ensure that the total amount of transfected DNA was equal in all wells (100 ng/well). Transfected COS-1 cells were exposed either to DMSO or 10 µM of rosiglitazone, and the luminescence activity of both luciferases, Fluc and Nluc, were assayed 24 h later using the Nano-Glo^®^ Dual Luciferase Assay System kit (Promega). This assay was performed four times independently, with two technical replicates in each assay.

#### 2.3.2. Characterization of mpFN26A[Fluc]/mpGL4.35[Nluc] Sensor System

To characterize mpFN26A[Fluc]/mpGL4.35[Nluc] sensor system, in uniplex and multiplex mode, COS-1 cells were exposed to a range of concentrations of PPARs’ reference agonists, 0.001, 0.01, 0.1, 1, 10, 100 and 10,000 nM of GW7647 (PPARα), 0.164, 0.410, 1.02, 2.56, 6.4, 16, 40, 100, 250, 1,000 and 10,000 nM of GW501516 (PPARβ), or 0.410, 1.02, 2.56, 6.4, 16, 40, 100, 250 1,000 and 10,000 nM of rosiglitazone (PPARγ). In these assays, cells were transfected with 40 ng of mpFN26A[Fluc/PPARα, -β and/or -γ] and 20 ng of mpGL4.35[Nluc], considering the results of the previous optimization assay. The luminescence activity of each luciferase was measured using Nano-Glo^®^ Dual Luciferase Assay System kit. These assays were independently performed three times, with two technical replicates in each assay. Uniplex mode refers to when the activity of only one PPAR is being measured, while multiplex mode refers to when multiple PPARs’ activities are being simultaneously measured. 

#### 2.3.3. Response of pBIND[Rluc]/pGL4.35[Fluc] Sensor System to PPARs’ Reference Agonists

In these assays, COS-1 cells were transfected individually (uniplex mode) or simultaneously (triplex mode) with pBIND[Rluc/PPARα, -β and/or -γ] and pGL4.35[Fluc]. In uniplex mode, cells were transfected with 40 ng of pBIND[Rluc/PPARα], pBIND[Rluc/PPARβ] and pBIND[Rluc/PPARγ], independently, while in multiplex mode 40 ng of a mixture of equal parts, i.e., 13.3 ng, of the three same vectors was used; plus 200 ng of the reporter vector pGL4.35[Fluc]. Cells were exposed either to DMSO (control) or each PPAR agonist: 10 µM GW7647 (PPARα), 10 µM GW501516 (PPARβ) or 10 µM rosiglitazone (PPARγ). The luminescent activity of both luciferases (Fluc and Rluc) was quantified using Dual-Glo^®^ Luciferase Assay System kit (Promega, Madison, WI, USA). This assay was independently performed three times, with two technical replicates per assay.

#### 2.3.4. Comparison of pBIND[Rluc]/pGL4.35[Fluc] and mpFN26A[Fluc]/mpGL4.35[Nluc] Systems

To compare the formerly used (pBIND[Rluc]/pGL4.35[Fluc]) and the new vector system (mpFN26A[Fluc]/mpGL4.35[Nluc]), their response to a range of rosiglitazone concentrations was evaluated. Due to differences in vectors size, the amount (ng) of mpFN26A[Fluc] and mpGL4.35[Nluc] was adjusted to ensure that the same number of vector units was present in each well during transfection, ensuring identical experimental conditions. COS-1 cells were cotransfected with 40 ng of pBIND[Rluc/PPARγ] and 200 ng of pGL4.35[Fluc], or with 44.5 ng of mpFN26A[Fluc/PPARγ] and 111.5 ng of mpGL4.35[Nluc], and exposed either to DMSO or to a range of rosiglitazone concentrations: 0.01, 0.1, 1, 10, 100, 1000 and 10,000 nM. The luciferases’ luminescence activity was measured 24 h after exposure to rosiglitazone with the corresponding kit, Dual-Glo^®^ Luciferase Assay System kit for the established system or Nano-Glo^®^ Dual Luciferase Assay System kit for the new system being tested. This assay was independently performed three times, with two technical replicates in each assay.

#### 2.3.5. Specificity of mpFN26A[Fluc]/mpGL4.35[Nluc] System

To evaluate the specificity of mpFN26A[Fluc]/mpGL4.35[Nluc], the sensor system was tested in uniplex and triplex modes, in transfected COS-1 cells exposed to DMSO or 200 μM of clotrimazole, a fungicide ligand of Pregnane X receptor (PXR) [42]. Luminesc ence activity was measured 24 h later with Nano-Glo^®^ Dual Luciferase Assay System kit (Promega, Madison, WI, USA). This assay was independently performed three times, with two technical replicates in each assay.

### 2.4. Culture of Cyanobacteria and Production of Crude Extracts and Fractions

Extracts were obtained from 96 cyanobacteria strains, selected from a variety of taxa, geographic origins, and community types to be screen with the biosensor developed. Twenty-eight strains were requested from the Blue Biotechnology and Ecotoxicology Culture Collection (LEGE-CC) [24] to produce biomass and the respective methanolic extract, and each crude extract was fractionated in 8 fractions following a standard methodology previously described [41], while 68 strains were requested from the Cyanobacteria Natural Products Library (LEGE-NPL) already fractionated, following the same methodology [41].

In detail, cyanobacteria were grown in 4 L propylene bottles under a light intensity of 10–30 µmol photons m^−2^s^−1^, 16 h:8 h (light:dark) at 25 °C and continuous aeration. Freshwater strains were cultivated in Z8 medium, and marine strains in Z8 medium supplemented with Tropic Marine™ salt (Tropic Marin^®^ REEF Premium REEF-Salt; 25 g/L; Tropical Marine Centre, Lisbon, PT) and B12 vitamin (Sigma Aldrich, Merck, St. Louis, MI, USA; 1 mL/L). Additionally, to induce stress and differentiation of heterocysts [43], 10 Nostocales strains were simultaneously cultivated in Z8 medium with and without nitrogen supplementation. The biomass of mature cultures was collected and stored at −80 °C until used. 

The cyanobacteria methanolic crude extracts (20 mg of each extract) were fractionated in a high-performance liquid chromatographer (HPLC; Waters e2695, Alliance-HPLC) equipped with an ACE C8 column (50 × 10 mm), the program consisting of a gradient of ultra-pure water and acetonitrile (10 to 100% acetonitrile; fractions A to C), followed by an isocratic elution at 100% acetonitrile for the fraction D to H (Figure S2) [41]. A 3.9 mL fraction was collected every 1.3 min, from minute 1 to minute 11.5, with an automatic sample collector into deep well plates. Collection times were established aiming to divide the samples’ mass in a uniform manner among fractions collected. This was experimentally executed using several crude extracts, of different cyanobacteria taxa and analyzing the dry weights of the fractions collected. Eight fractions (A to H) were collected from each strain, dried, resuspended in DMSO at a final concentration of approximately 5 mg/mL, and stored at −80 °C until used.

#### Screening of PPARs’ Ligands in Cyanobacteria Fractions

The newly developed mpFN26A[Fluc]/mpGL4.35[Nluc] sensor system was used to perform the primary screening of 848 cyanobacteria fractions for PPARs’ ligands in triplex mode. COS-1 cells were exposed either to DMSO (two replicates per plate), to each reference PPARs’ agonists [10 µM WY14643 (PPARα), 10 µM GW501516 (PPARβ) and 10 µM rosiglitazone (PPARγ)—two replicates of each per plate], or 1 µL (~5 ng/µL) of each cyanobacteria fraction (one replicate per plate). Luminescence activity of Nluc and Fluc was measured 24 h later using the Nano-Glo^®^ Dual Luciferase Assay System kit. 

Fractions that presented a Log2 transformed fold induction (FI) value below -1 or above 1 were considered hits and were selected to be retested with the same sensor system in triplex mode to confirm the results. Confirmed hits were then subjected to another validation/elucidation procedure, using the same sensor system but in uniplex mode, to access the effect of the samples in each PPAR individually. In this final assay, cells were transfected with 40 ng of mpFN26A[Fluc/PPARα, -β or -γ], and 20 ng of mpGL4.35[Nluc], and exposed either to DMSO, to 1 µL (5 ng/µL) of a cyanobacteria fraction considered hits in the latter multiplex screening, or to the reference agonist of each PPAR: 10 µM WY14643 (PPARα), 10 µM GW501516 (PPARβ) or 10 µM rosiglitazone (PPARγ). Luminescence activity of Nluc and Fluc was quantified using the Nano-Glo^®^ Dual Luciferase Assay System kit. This assay was independently performed three times, with two technical replicates in each assay.

### 2.5. Data Analysis and Statistics

Fold induction values observed were calculated relatively to DMSO control by dividing the raw luminescence signal observed with Fluc, by the raw luminescence observed with Rluc, in the pBIND[Rluc]/pGL4.35[Fluc] system; or Nluc raw luminescence signal divided by the Fluc raw luminescence in the mpFN26A[Fluc]/mpGL4.35[Nluc] system; and then normalized/divided by DMSO fold induction (FI) value determined in each plate. The fluorescence ratios analyzed are between the luminescence read with the responsive vector and the constitutively expressed vector of each system, either Fluc/Rluc in the initial system or Nluc/Fluc in the developed sensor system, and then normalized by the same ratio observed in the solvent (DMSO) control samples, this last normalization giving rise to FI values. In the screening assays, the resulting values were Log2 transformed to achieve data normality and presented as the mean and the standard error of the mean (SEM). Values > 1 represent induced PPARs activity relatively to control, while values < −1 represent inhibited activity relative to control. One-Way Analysis of Variance (ANOVA) followed by Dunnett’s test was used to determine significant differences between sample results and the control (*p* < 0.05), after verifying the parametricity of data. Statistical analysis and plots were performed on SigmaPlot v. 12.5.

The half-maximal effective concentration (EC_50_), the limit of detection (LOD), the limit of quantitation (LOQ), the linear range of detention (LRD) [44,45,46,47]) and the precision of the regression analysis [standard error (SE) of the response estimate, SEy] were computed to characterize the mpFN26A[Fluc]/mpGL4.35[Nluc] sensor system in uniplex and multiplex modes and to compare it with the previous system in use. EC_50_ of the reference PPAR agonists was calculated by fitting the sensor response to a range of nine Log agonist concentrations, to the dose-response sigmoid four-parameter Hill logistic equation [48], using the freely available software Dr Fit (sourceforge.net/projects/drfit) [49]. EC_50_ was taken as an indicator of sensor’s sensitivity; the lower the EC_50_, the lower the concentration of a drug required to produce 50% of the maximum sensor signal and so the higher the sensitivity of the sensor. LRD was defined as the Log concentration range of the sigmoid curve where FI intensity is proportional to the Log concentration of the PPAR reference agonist analyzed; it corresponds to the Log concentration range where the relationship between these two parameters is linear. SEy was calculated for uniplex and triplex modes, using the SE of the calibration curve, in the section where it contains samples in the range of the LOQ, obtained from a regression analysis of the linear section of the dose-response curve. LOD was calculated as 3.9 × SEy/slope of LRD and LOQ as 3.3 × LOD [44]. These are also parameters of the biosensor’s sensitivity.

The quality of the results obtained in the screening assays was assessed by computing the Z-factor value [50,51] for each of the 11 96-well plates used in the various screening assays, based on the median absolute deviation and the relative standard deviation of the results obtained for each of the three PPARs’ reference agonists in the same well-plates. Plates were classified into four categories (Table S2) according to their Z-factor [51]. Plates with Z-values smaller than 0.5, for all three reference agonists, were not considered trustworthy and were discarded.

In the final hit confirmation assays in uniplex mode, FI values were considered hits if the Log2 transformed mean value of a fraction was higher than 1 or smaller than one; or if the SEM of the log2 transformed values crossed one of the threshold lines defined for hit values: X > 1 and X < −1.

## 3. Results and Discussion

### 3.1. Optimization of Vector Ratio in mpFN26A[Fluc]/mpGL4.35[Nluc] System 

**Figure 2:**
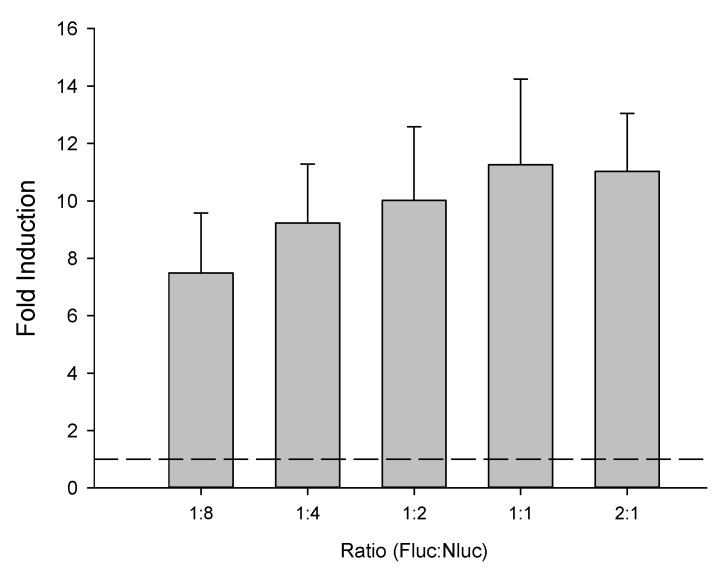
Transactivation activity (fold induction) values observed on cells transfected with different proportions of mpFN26A[Fluc/PPARγ] and mpGL4.35[Nluc] vectors, challenged with 10 µM rosiglitazone. The basal response to the control (DMSO) is represented by the dashed line (y = 1). Data are shown as mean ± standard error of the mean (SEM) of four assays performed independently (n = 4). DMSO concentration did not exceed 0.1% per well.

The optimal proportion between the two vectors of the mpFN26A[Fluc]/mpGL4.35[Nluc] sensor system was analyzed, considering the fold induction values (FI) values obtained with different vector ratios. Fluc stands for firefly luciferase and Nluc for Nanoluc^®^ luciferase. FI refers to the luminescence ratio observed between both luciferases of a vector system Nluc/FLuc or Fluc/Rluc of each sample, expressed relatively to the same ratio observed in the DMSO controls in each plate. This procedure converts raw data into normalized data. The values observed for the various vector ratios analyzed were not statistically different (One-Way ANOVA, Tukey test; *p* = 0.866), despite showing a trend towards a plateau when mpFN26A[Fluc] to mpGL4.35[Nluc] proportion equaled (1:1) or exceeded (2:1) (Figure 2).

The ratio 2:1 was chosen for subsequent assays since, at this ratio, fold induction values are not dependent on the ratio between vectors—from the ratio 1:1 onwards the sensor’s signal is independent of the vector ratio. Additionally, this double quantity of constitutive vector provides a higher amount of synthesized GAL4-DBD/PPAR-LBD fusion protein to be rendered available for possible PPARs ligands, leading to an increase in the range of detention even when ligands are present at extreme concentrations.

### 3.2. Characterization of mpFN26A[Fluc]/mpGL4.35[Nluc] Sensor System

The dose-response curves (Figure S3) and various performance parameters (Table 1) were determined and analyzed to characterize the new mpFN26A[Fluc]/mpGL4.35[Nluc] biosensor system, using COS-1 cells exposed to broad concentration ranges of three PPARs agonists, in uniplex and triplex modes. Uniplex mode refers to when the activity of only one PPAR is being measured, while multiplex mode refers to when multiple PPARs are being measured simultaneously. 

EC_50_ could only be determined for data obtained in uniplex mode, since only in this mode does the effect concentration data follow a sigmoid model. In multiplex mode, the sensor response to PPARs agonists did not reach the maximal values (plateau) for the concentrations tested, and data possibly did not follow a monophasic model, which precluded fitting the Hill equation to the dose-response curves of the results obtained. In uniplex mode, EC_50_ was the lowest for GW501516 (PPARβ), 3.6 nM, and the highest for rosiglitazone (PPARγ), 29.4 nM (Table 1 and Figure S3). The higher concentrations of the reference agonists used seem to become toxic to the biosensor-expressing cells since a decrease in the raw luminescence value of the constitutively expressed vectors mpFN26A[Fluc] and pBind[Rluc] was observed (Table S3). They deviate data from the Hill model, prohibiting reliable readouts and so could not be used to attempt to reach the model plateau.

The SEy of the regression at the LRD was similar in uniplex and triplex modes, and also when comparing the three standard agonist compounds tested—ranging from 5.02 to 11.04 nM (Table 1)—which indicates the stability of the biosensor signal irrespective of the mode used and the sample assayed. LOD varied between 0.36 nM for rosiglitazone detection in uniplex mode and 1.35 nM when detecting GW501516 in triplex mode. LOQ varied between 1.18 nM for rosiglitazone in uniplex mode and 4.44 nM for GW501516 in triplex mode. The results tend to be higher in multiplex mode possibly due to eventual low-affinity dual- or pan-agonism of the tested ligands. In that scenario, ligands would bind, not only to the LBD of the PPAR they are highly specific to but also to the LBD of the other two PPARs albeit with less affinity, causing a reduction of LOD and LOQ values. 

Comparing our results with the available bibliography, rosiglitazone’s LOQ in uniplex mode is of the same range as that observed using LC-MS in rat plasma and tissues, 1.68 nM [52]. In addition, for rosiglitazone, the EC_50_ observed with the sensor in uniplex mode for PPARγ, 29.37 nM, was in the same range as determined with pBIND[Rluc]/pGL4.35[Fluc] system by other authors [53]. EC_50_ values obtained with PPARα for GW7647, and PPARβ for GW501516 in uniplex mode in stable cell lines expressing the respective LBDs, were also in the range of those obtained in the present work [54].

The LRD was narrower in uniplex mode, ranging from two to three orders of magnitude, while in triplex mode, the system presented four orders of magnitude range or broader for the three PPARs agonists tested (Table 1). A broader LRD is beneficial for screening since the concentration of the bioactive compounds in the fractions and subtractions of an extract to be analyzed is unknown, and the higher the LRD the higher the probability of bioactive compounds concentration falling inside the LRD during screening.

### 3.3. Comparison of pBIND[Rluc]/pGL4.35[Fluc] and mpFN26A[Fluc]/mpGL4.35[Nluc] Systems

In triplex mode, the newly developed mpFN26A[Fluc]/mpGL4.35[Nluc] sensor system delivered higher induction values than those of the departure sensor system pBIND/pGL4.35[Fluc] in the same mode (Table S4). The two systems were compared by exposure to 10 μM of a reference agonist of each PPAR. In uniplex mode, mpFN26A[Fluc]/mpGL4.35[Nluc] sensor delivered higher fold induction values only for GW7647 and lower for GW501516 and rosiglitazone (Table S4).

Both sensor systems were compared in an assay where cells were transfected with the same quantity of each vector (number of vector copies) in uniplex mode and exposed to a range of rosiglitazone concentrations (Table S3). The raw luminesce of the signal of both vectors was higher in the mpFN26A[Fluc/PPARγ]/mpGL4.35[Nluc] sensor system than in the departure system, rendering a more intense signal. The latter system also features a higher sensitivity inferred from the lower LOD and LOQ values (Table 1). On the other side, in uniplex mode, the mpFN26A[Fluc]/mpGL4.35[Nluc] system features a lower LRD. 

### 3.4. Specificity of mpFN26A[Fluc]/mpGL4.35[Nluc] System

The specificity of the mpFN26A[Fluc]/mpGL4.35[Nluc] sensor system was evaluated in uniplex and triplex modes when challenged with 200 μM of clotrimazole (Table 2), an exogenous ligand of PXR. This fungicide did not cause a significant statistical effect on the system’s FI when compared to the DMSO control, neither in uniplex nor in triplex modes. PXR belongs to the same subfamily of NRs as PPARs—the Thyroid Hormone Receptor-like subfamily, whose primary function is to sense the presence of sterols and foreign toxic substances, promoting their clearance from the organism [42]. According to this result, it is possible to be assumed that our system had specificity for PPARs ligands. 

### 3.5. Screening of PPARs Ligands in Cyanobacteria Fractions

**Figure 3:**
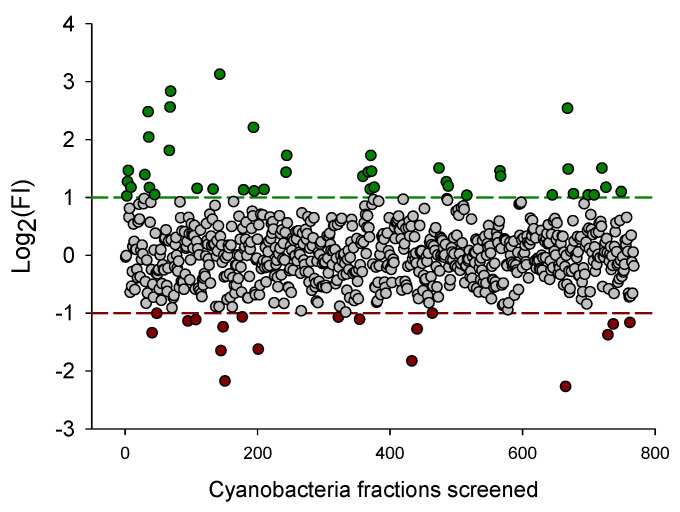
Log_2_ transformed fold induction (FI) values of the 768 cyanobacteria fractions screened with the mpFN26A[Fluc]/mpGL4.35[Nluc] sensor system, in triplex mode, that passed the quality control analysis. Red dots (n = 18) correspond to fractions featuring inhibition (Log2 FI *≤* −1), green dots to fractions showing induction (Log2 FI *≥* 1) (n = 42), and grey dots correspond to fractions showing no induction nor inhibition (-1<Log2 FI *≤* 1) of PPARs activity (n = 708). Dimethyl sulfoxide (DMSO) concentration did not exceed 0.5 % per well.

The primary screening of 848 cyanobacteria fractions, using the mpFN26A[Fluc]/mpGL4.35[Nluc] sensor system in triplex mode, made use of 11 96-well plates. Those results underwent a quality control (QC) procedure that resulted in the exclusion of plate #11 (Z-values ≤ 0.5). This plate did not pass the QC for any of the three PPARs reference agonists assayed, and for that, it was waived (Table S5). Of the 768 cyanobacteria fractions of the remaining 10 plates that passed the QC, 60 fractions were classified as hits (7.8%) (Figure 3). Of those, 42 fractions (5.47%) featured FI activity above 2.0, a threshold defined to correspond to induction activity, and 18 fractions (2.34%) presented FI values below 0.5, corresponding to repression activity. The threshold values 2 and 0.5, correspond to −1 and 1 when FI values were Log2 transformed (Figure 3).

**Figure 4:**
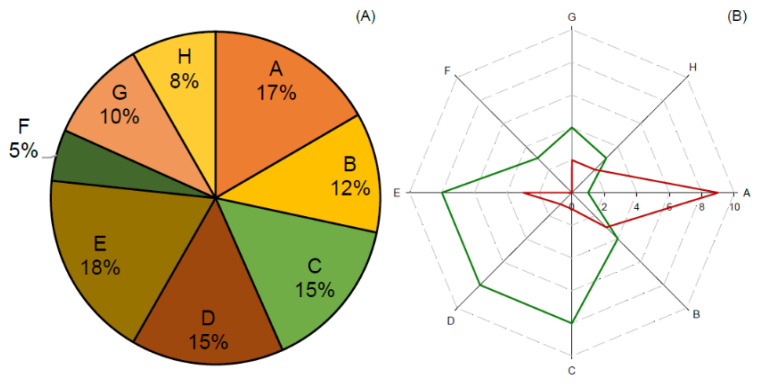
Results of the primary screening hits. Hits distribution (%) among the different cyanobacteria crude extract fractions, from A to H, corresponding A to the most polar fraction and H to the least polar (**A**). The number of hits causing repression [fold induction (FI) ≤ 0.5; red line] or induction (FI ≥ 2; green line) on PPARs activity (**B**).

The fractions that presented a higher number of hits were fractions E, A, C and D, while a lower number of hits was observed in fractions H and F (Figure 4A). Since fractioning of crude extracts was performed using reverse phase chromatography, with water and acetonitrile, from fractions A to C the polarity of the eluent mixture decreases, and so the polarity of the eluted compounds. The most polar compounds were eluted in the initial samples, from A to C, where 44% of the hits occurred, while less polar compounds were eluted in the fractions D to H, where 56% of the hits were collected. Fraction A presented the highest number of repression hits (9), while fractions C, D and E presented the highest number of induction hits, 8 hits in each fraction (Figure 4B).

The values of the repression hits varied between 0.206 and 0.500 and those of the induction hits between 2.037 and 8.746 (Table S6). The 60 hits observed occurred in 37 Cyanobacteria strains of different orders: 15 Synechococcales, 12 Nostocales, 4 Oscillatoriales, 3 Chroococcales, 1 Pleurocapsales and 2 in non-identified strains (Table S6). 

The hits identified were assayed again in the secondary screening, using the same sensor system in triplex mode to confirm the obtained results. Six of those fractions (10%) maintained their PPAR modulation activity (Table 3). Of those, four fractions induced PPARs’ activity (FI ≥ 2.0) and two fractions inhibited activity (FI ≤ 0.5). The observed reduction in hit number could be due to the single replicate layout of the initial screening plates. It should be noted that standard transactivation assays use in general a higher number of technical and biological replicates, as used here for the characterization of the novel mpFN26A[Fluc]/mpGL4.35[Nluc] system. Contrarily, medium and high throughput screening assays do not use replicates [50,51] due to the high costs involved, and thus, confirmatory assays are crucial to validate observed hits. Nonetheless, the two-step validation allows for improved screening efficiency and celerity, while maintaining hit reliability.

**Figure 5:**
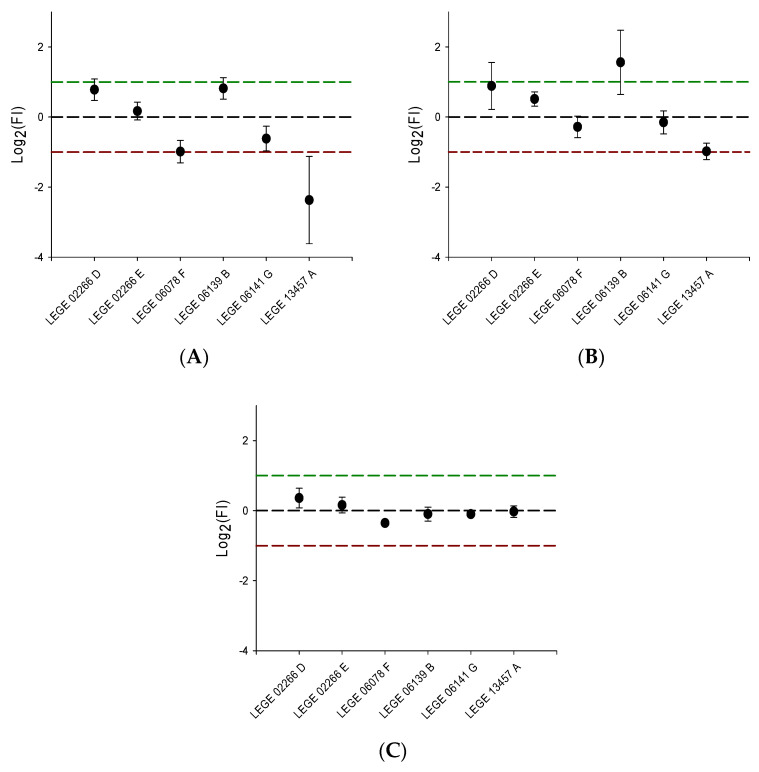
Transactivation activity observed in cells transfected with mpFN26A[Fluc]/mpGL4.35[Nluc] sensor system, in uniplex mode in PPARα (**A**), PPARβ (**B**), and PPARγ (**C**). These six cyanobacteria fractions were considered hits in the two previous triplex assays using the same sensor system. Data above and below the threshold lines represent hits, with values y ≥ 1 (green line) corresponding to induction and y ≤ −1 (red line) to repression. The basal response to the solvent control (dimethyl sulfoxide, DMSO; concentration not exceeding 0.5 % per well) is represented by the black dashed line (y = 0). Data are shown as mean ± standard error of the mean (SEM) (n = 3).

The six cyanobacteria fractions that confirmed their activity to transactivate PPARs in the secondary screening assay were then assayed in uniplex mode to determine which PPAR isotype or isotypes were being targeted by the bioactive compounds of each cyanobacteria fractions (Figure 5). Fractions D of LEGE 02266 and B of LEGE 06139 simultaneously activated PPARα and PPARβ, while fraction A of LEGE 13457 simultaneously inhibited PPARα and PPARβ. On the other hand, fraction F of LEGE 06078 showed specific inhibition of PPARα activity. None of the fractions presented substantial induction or inhibition activity on PPARγ. In short, seven hits were confirmed in uniplex mode from extracts of four strains: LEGE 02266, LEGE 06139, LEGE 13457 and LEGE 06078; three extracts simultaneously presented a dual agonist action over PPARα and -β. Further analysis should be carried out to isolate the compounds responsible for the sensor’s signal and characterize the compounds with agonistic/inverse agonistic activity. 

Three of the four strains whose fractions presented PPARs’ modulatory bioactivity had been previously signaled as producers of bioactive metabolites within the scope of other projects. Those strains are *Nodosilinea (Leptolyngbya) antarctica* LEGE 13457, which was reported to produce carotenoids with the potential to be used in the treatment of psoriasis, due to their anti-inflammatory activity [55]; *Oxynema acuminatum* LEGE 06078, which presents compounds with genotoxic activity [56,57]; and *Cyanobium* sp. LEGE 06139, whose compounds showed moderate toxicity in RKO, a colon carcinoma cell line [58]. Some of those results may be related to the PPARs’ transactivation activity here reported, since genes known to be under PPARs’ control are related to inflammation and tumorogenesis, among many others. Since the bioactive compounds are not yet isolated nor identified, other tools must be used to obtain additional information.

### 3.6. Low Firefly Luciferase (Fluc) Raw Values Observed with Some Fractions Assayed

During the optimization assays, it was observed that the constitutive vector of both biosensor systems, which simultaneously encodes the hybrid GAL4-DBD/PPARx-LBD protein and a control luciferase (Rluc or Fluc for pBIND or mpFN26A, respectively), presented a diminished transcription activity at ligand concentrations above 100 nM (Table S3). Those reporter genes are constitutively expressed by the vectors pBind[Rluc] and mpFN26A[Fluc] and their expression is independent of the PPARs’ modulation activity through transactivation. We hypothesize that this reduction in transcription activity is due to a general toxic effect of the PPAR ligand on the biosensor’s cells at higher concentrations. As such, the expression of those constitutive vectors, assessed by the raw luminescence of the respective reporter gene, may possibly be used as a proxy of sample toxicity, in addition to the use they already have in quantifying the transfection rate. 

This hypothesis needs further analysis and confirmation in future studies, but seems supported by previous works. Rosiglitazone, for instance, was shown to inhibit adrenocortical cancer cell proliferation, in a dose- and time-dependent manner, by interfering with the IGF-IR intracellular signaling at similar concentrations, as evaluated by different techniques (MTS, thymidine uptake and cell counting), with a calculated IC50 of 22.48 μM [59]. Rosiglitazone concentrations above 10 μM were found to decrease both mRNA and protein levels of LRP1, explained mechanistically, by the downregulation of PPARγ in a time- and concentration-dependent manner (40%), being the remaining loss of LRP1 (60%) attributed to degradation in the lysosomal system [60].

**Figure 6:**
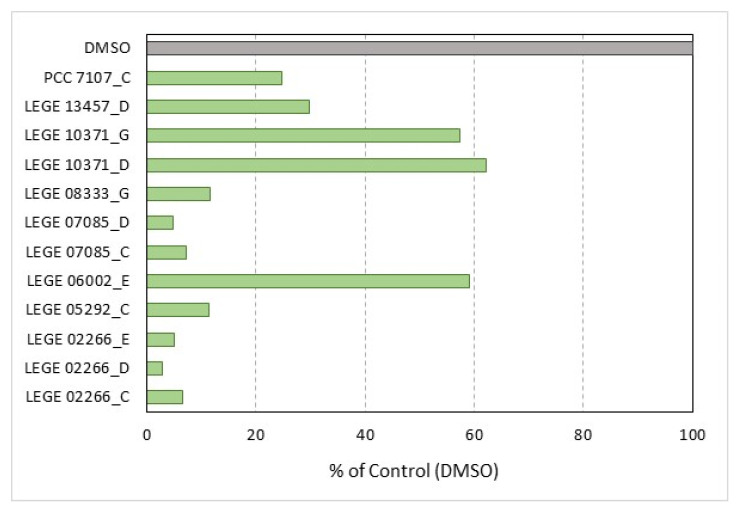
Firefly luciferase (Fluc) luminescence values observed in cells exposed to 12 specific fractions of 8 cyanobacteria strains. These values were particularly low when compared to solvent control (DMSO). Cells were transfected with the constitutively expressed vectors mpFN26A[Fluc/PPARα], mpFN26A[Fluc/PPARβ] and mpFN26[Fluc; PPARγ] and pGL4.35[Nluc]. DMSO concentration did not exceed 0.5 % per well. Results are shown as percentage DMSO luminescence (100%).

During the screening, 12 fractions of 8 strains presented particularly lower Fluc raw values (Figure 6): *Sphaerospermopsis* sp. LEGE 02266, *Phormidium* sp. LEGE 05292, *Cyanobium* sp. LEGE 06002, *Nodosilinea* sp. LEGE 07085, *Pseudanabaena* cf. *curta* LEGE 10371, *Nodosilinea (Leptolyngbya) antarctica* LEGE 13457, unidentified unicellular Synechococcales LEGE 08333 and *Nostoc* sp PCC 7107. Those results were obtained mostly with the fractions C (33%) and D (33%), followed by fractions E (17%) and G (17%). On the other hand, fractions A, B, F and H did not show low Fluc raw values. Summing up, presumed toxicity detected by the system, based only on Fluc raw values, was observed on fractions with intermediate polarity, and it was not found in very polar on non-polar fractions. In fact, several compounds present in cyanobacteria fractions with intermediate polarity were identified as peptides and depsipeptides with great anticancer potential [58]. 

The strains identified to have low Fluc raw luminescence were looked at for the presence of cyanotoxins in the genus, but its presence is not documented [24], which may indicate that the toxicity is due to other compounds than cyanotoxins. The fraction C of *Phormidium* sp. LEGE 05292 has been used as a positive control for cytotoxicity in other projects of our research team since it is known to contain portoamides A and B, which are cytotoxic peptides [41,61]. Additionally, *Nodosilinea (Leptolyngbya) antarctica* LEGE 13457 induced apoptosis in the HaCaT cell line, a human epidermal keratinocyte line [55]. 

Reduced Fluc values were previously referred, during the screening of low molecular weight compound libraries, and related to either toxicity or interference with Fluc activity [36]. For example, flavonoids are identified as one group of compounds that can inhibit Fluc [62] and are present in cyanobacteria [55]. However, in our case, reduced raw values were observed in both enzymes used in the constitutive vectors of the two sensor systems, Fluc and Rluc, when exposed to rosiglitazone concentrations above 10–100 nM. Nonetheless, more research is needed to fully understand the origin of the luminescence decrease, and it must be carefully looked at when analyzing screening results, because it may interfere with hits detection, originating false positives and negative hit results.

## 4. Conclusions

The major improvements of the new whole-cell biosensor developed included the use of different reporter genes to increase signal intensity and stability, a different promoter to modulate the expression of Fluc, and multiplexing the assay to increase efficiency and information obtained per unit of effort. Additionally, multiplexing allowed for the detection of fractions with possible specific, dual- or pan-agonist compounds, and to evaluate synergic action in more than one PPAR. The use of different luciferases and promoters rendered a more luminous assay with higher sensitivity, with limit of detection (LOD) ranging from 0.36 to 0.89 nM in uniplex mode, and 0.89 to 1.34 nM in multiplex mode, for the various reference PPARs agonists used. In triplex mode, the new biosensor featured a broader dynamic range which is beneficial for screening, despite featuring a lower LOD. Sensor specificity was checked with clotrimazole. The utility of the developed sensor was validated by the identification of fractions of cyanobacterial extracts that contained PPAR agonist properties, by conducting a medium-size screening trial with 848 fractions of 96 different cyanobacterial strains. Primary and secondary screening assays were performed using the sensor in multiplex mode and final hits were reassayed in uniplex mode to determine which PPARs were being targeted by the bioactive extracts. In the end, seven hits were confirmed in uniplex mode, on fractions of four strains: LEGE 02266, LEGE 06139, LEGE 13457 and LEGE 06078, three extracts simultaneously presenting a dual agonist action over PPARα and -β. 

## Figures and Tables

**Table 1:** Performance parameters determined for mpFN26A[Fluc]/mpGL4.35[Nluc] sensor system in uniplex and triplex modes, when reading different concentrations of PPARα, -β or -γ agonists (GW7647, GW501516 or rosiglitazone, respectively). The performance parameters obtained for pBIND[Rluc]/pGL4.35[Fluc] system in uniplex mode, when challenged with rosiglitazone, are also presented for comparative purposes in the grey background column.

Compound		GW7647 (PPARα Agonist)		GW501516 (PPARβ Agonist)		Rosiglitazone (PPARγ Agonist)		
Mode		Uniplex	Triplex	Uniplex	Triplex	Uniplex	Triplex	Uniplex pBIND/pGL4.35
**EC_50_ (nM)**		12.97	-	3.59	-	29.37	-	-
**Linear equation** Parameters *	***a***	7.565	−3.084	20.31	1.541	−35.43	−30.94	−3.084
	***b***	28.71	24.86	41.20	24.59	54.83	32.87	24.86
**R^2^**		0.948	0.981	0.929	0.933	0.941	0.912	0.980
**SEy response (nM)**		8.631	5.657	9.416	8.482	5.017	11.04	5.656
**LOD (nM)**		1.173	0.887	0.891	1.345	0.357	1.310	0.887
**LOQ (nM)**		3.870	2.928	2.942	4.439	1.178	4.322	2.928
**LRD (nM)**		[1;1000]	[1;10,000]	[0.4;100]	[1;10,000]	[6.4;100]	[1.6;10,000]	[10;1000]

* On the linear equation parameters, *a* corresponds to the ordinate at the origin and *b* to the slope. This linear equation was determined in the range of concentration values where fold induction is proportional to the log concentration of each PPAR agonist. The coefficient of determination (R^2^) refers to the goodness-of-fit of the linear model to data and SEy to the standard error of the linear model estimate.

**Table 2:** Fold induction values observed with the mpFN26A[Fluc]/mpGL4.35[Nluc] sensor system, in uniplex and triplex modes, when exposed to solvent control (DMSO) or to 200 μM of clotrimazole. Data are shown as mean ± standard error of the mean (SEM) (n = 3). The *p* value resulted from the statistical analysis (One-Way ANOVA; Tuckey test; *p* < 0.050), relatively to the solvent control. Dimethyl sulfoxide (DMSO) concentration did not exceed 0.1% per well.

	Uniplex			Triplex
	PPARα	PPARβ	PPARγ	PPARα, -β and -γ
**DMSO**	0.987 ± 0.025	1.026 ± 0.030	0.975 ± 0.040	1.042 ± 0.010
**Clotrimazole**	0.944 ± 0.051	1.167 ± 0.085	0.976 ± 0.114	1.15 ± 0.35
***p*-value**	0.871	0.156	0.239	0.574

**Table 3:** Transactivation activity values observed during the secondary screening assays, using cells exposed to fractions of cyanobacteria strains formerly identified as hits in the primary screening, using the mpFN26A[Fluc]/mpGL4.35[Nluc] sensor system in triplex mode. Fold induction (FI) values ≤ 0.5 were considered repressed activity (red), while FI ≥ 2 were considered inducted PPAR activity (green). Dimethyl sulfoxide (DMSO) concentration did not exceed 0.5 % per well.

Order	Strain	Taxon	Fraction	FI Confirmation
**Nostocales**	LEGE 02266	*Sphaerospermopsis * sp.	D	2.448
			E	2.420
**Oscillatoriales**	LEGE 06078	*Oxynema acuminatum*	F	3.003
	LEGE 06139	*Cyanobium * sp.	B	2.229
	LEGE 06141	*Oculatella * sp.	G	0.494
	LEGE 13457	*Nodosilinea antarctica*	A	0.489

## Data Availability

Data available as supplementary material. Otherwise, it can be requested from the corresponding authors.

## Supplementary Materials

The following supporting information can be downloaded at: https://www.mdpi.com/article/10.3390/s23031338/s1, Figure S1: Schematic representation of vectors constructed for the new biosensor and original vectors where parts were taken from for their construction; Table S1: List of primers used; Figure S2: Details on the high-performance liquid chromatographer program used to fractionate the cyanobacteria methanolic crude extracts; Table S2: Z-factor interpretation based on Goktug et al. 2013 [50]; Figure S3: Luminescence response observed with mpFN26A[Fluc]/mpGL4.35[Nluc] sensor system in uniplex and triplex modes. Table S3: Raw luminescence values of the reporter genes of the vectors used; Table S4: Transactivation activity observed with both sensor systems used; Table S5: Z-factor values determined for the 11 96-well plates of the primary screening; Table S6: Heat map showing the of transactivation activity observed in cells upon exposure to fractions of various cyanobacteria strains in the primary screening.
