# Supplementary material for: A Multiplex Molecular Cell-Based Sensor to Detect Ligands of PPARs: An Optimized Tool for Drug Discovery in Cyanobacteria
Source: Sensors (Basel). 2023 Jan 25;23(3):1338. doi: 10.3390/s23031338 (PMC9919141; doi:10.3390/s23031338)
Supplement: Supplementary file 1 [file sensors-23-01338-s001.zip › sensors-2116632-supplementary.pdf]

SUPPORTING INFORMATION

A multiplex molecular cell-based sensor to detect ligands of PPARs: an optimized tool for drug discovery in cyanobacteria

Inês Páscoa [1¥], Rita Biltes [1,2,3¥], João Sousa [1], Marco Preto [1], Vitor Vasconcelos [1,3], Luís Filipe Castro [1,3], Raquel Ruivo [1] and Isabel Cunha [1*]

[1] CIIMAR/CIMAR - Interdisciplinary Centre of Marine and Environmental Research, University of Porto, 4450-208 Portugal

[2] ICBAS - Instituto de Ciências Biomédicas Abel Salazar, University of Porto, 4050-313 Portugal

[3] FCUP - Faculty of Sciences, Department of Biology, University of Porto, 4169-007 Portugal

*Correspondence: IC - isabel.cunha@ciimar.up.pt; (+351) 223 401 800

[¥]Shared first authorship

Figure S1: Schematic representation of the vectors constructed for the new biosensor, including the original vectors where parts were taken from for their construction. The vector mpFN26A (A) was constructed with parts of pFN26A[luc2\TK] and pGL4.54 and, the vector mpGL4.35 (B) was constructed with parts of pGL4.35 and pNL1.1[Nluc]. All vectors were acquired to Promega. This procedure was executed by NZYTech according to our instructions.

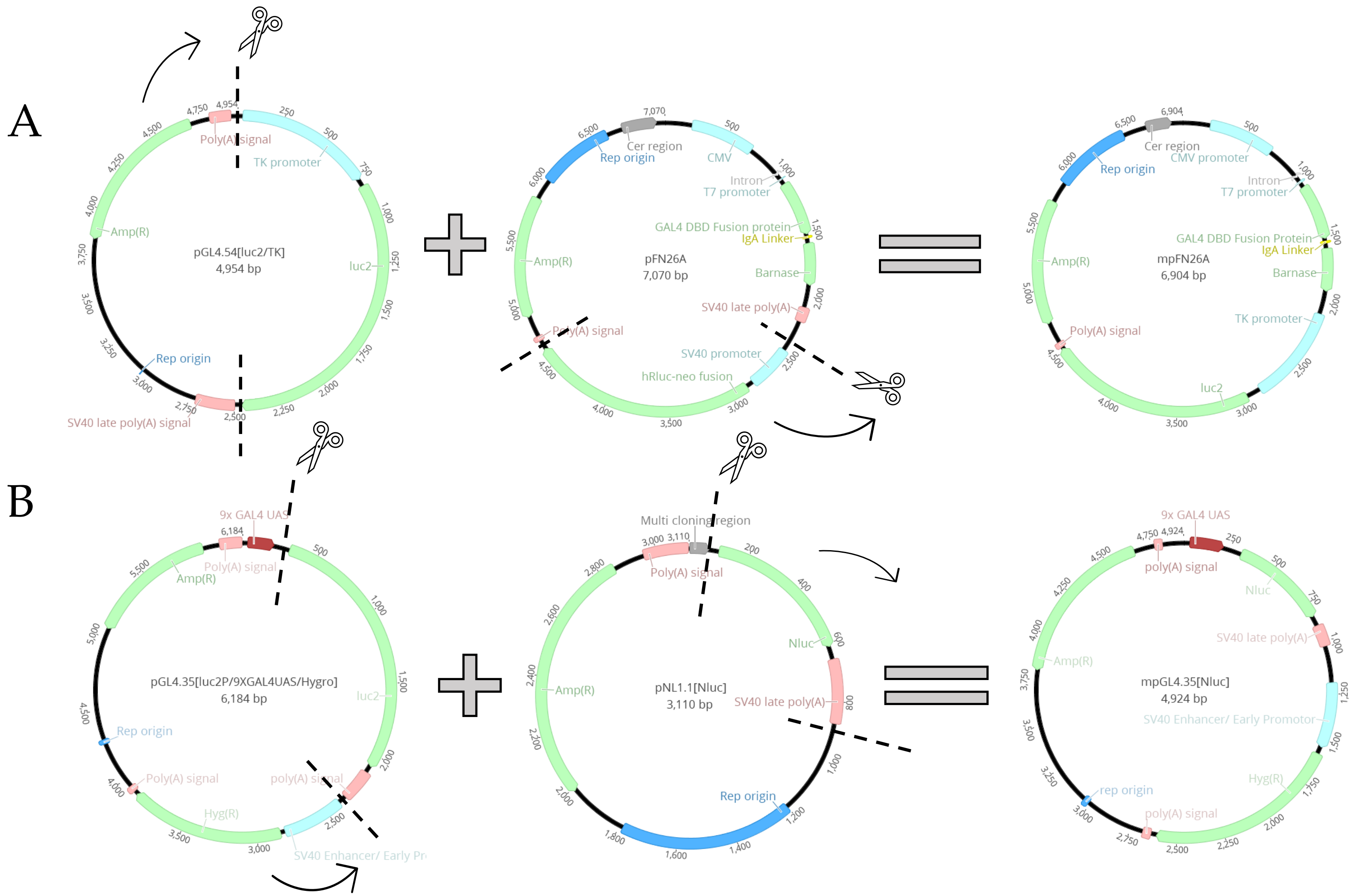

Table S1: List of primers used. Sequences of the primers used to amplify the hinge and ligand binding domain (LBD) of *Homo sapiens* PPARα, -β and –γ, and specific restriction enzymes used to insert the resulting PCR products into the respective pBIND or mpFN26A vectors.

PPAR	Vectors	Enzymes	Primers' Sequence
PPARa	pBind	XbaI	F:5′-CCCTCTAGAATGTCACACAACGCGATT-3′
		KpnI	R:5′-ATAGGTACCTCAGTACATGTCCCTGTAGA-3′
	mpPFN26A	SgfI	F:5′-CGATAGCGATCGCCATGTCACACAACGCGATT-3′
		PmeI	R:5′-CGTTTAAACTCAGTACATGTCCCTGTAGA-3′
PPARb	pBind	XbaI	F:5′-CCCTCTAGAATGTCACACAACGCTATC-3′
		KpnI	R:5′-ATAGGTACCTTAGTACATGTCCTTGTAGATC-3′
	mpPFN26A	SgfI	F:5′-CGATAGCGATCGCCATGTCACACAACGCTATC-3′
		PmeI	R:5′-CGTTTAAACTTAGTACATGTCCTTGTAGATC-3′
PPARg	pBind	BamHI	F:5′-GCTGCTGGATCCGAATGCCACAGGCCGAGAAGGAG-3′
		KpnI	R:5′-ATAGGTACCCTAGTACAAGTCCTTGTAGATCTCC-3′
	mpPFN26A	SgfI	F:5′-CGATAGCGATCGCCATGCCACAGGCCGAGAAGGAG-3′
		PmeI	R:5′-CGTTTAAACCTAGTACAAGTCCTTGTAGATCTCC-3′

Figure S2: Details on the high-performance liquid chromatographer program used to fractionate the cyanobacteria methanolic crude extracts, consisting of a gradient of ultra-pure water and acetonitrile (10 to 100% acetonitrile), followed by isocratic elution at 100% acetonitrile, during 14 min [41]. Eight fractions (A to H) were collected from each strain.

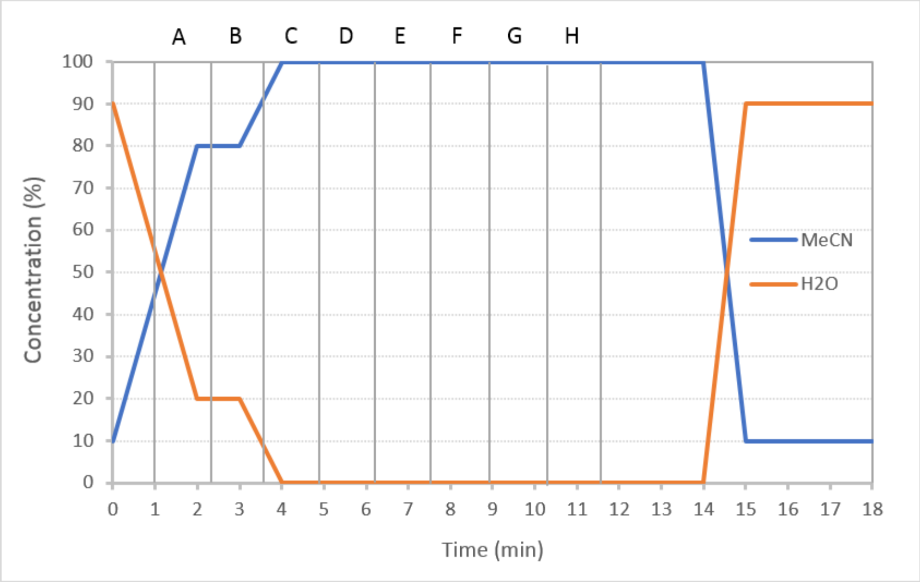

Table S2: Z-factor interpretation based on Goktug et al. 2013 [50].

Z-factor value	Screening interpretation
**1**	Ideal/perfect assay, assay validated
**]0.5; 1]**	Excellent assay, assay validated
**]0.0; 0.5]**	Marginal assay, assay validated
**< 0**	DMSO variation and samples signal overlapped, invalid assay

Figure S3. Luminescence response observed with mpFN26A/mpGL4.35[Nluc] sensor system in uniplex and triplex modes. Values observed after exposure to different concentrations of PPARα, -β or -γ agonists (GW7647, GW501516 or rosiglitazone, respectively). Dose-response curves (full lines) and EC_{50} values (dashed lines) are shown only for the uniplex mode.

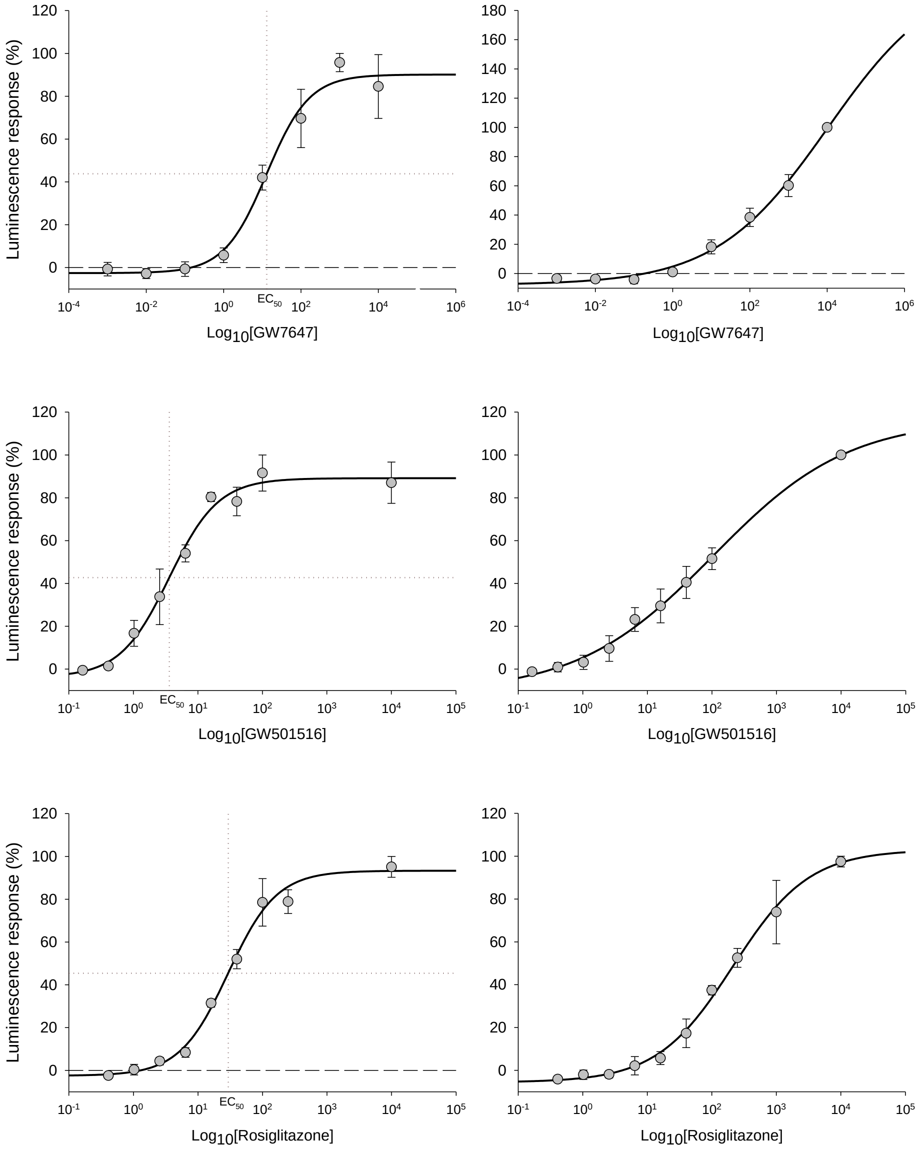

Table S3. **Raw luminescence values of the reporter genes of the vectors used.** Luminescence values of firefly (Fluc) and Renilla (Rluc), and Nanoluc® (Nluc) and firefly luciferase, observed with pBIND[Rluc]/pGL4.35[Fluc] and mpFN26A[Fluc]/ mpGL4.35[Nluc] sensor systems, respectively. Cells were exposed to the solvent control (DMSO; not exceeding 0.1 % per well) and a gradient of rosiglitazone concentrations. Data are shown as mean ± standard error of the mean (SEM) (n=3). Black arrows indicate decreased transcription activity of pBind[Rluc] and pFN26A[Fluc] at higher rosiglitazone concentrations.

		**pBIND/pGL4.35[Fluc] Sensor**		**mpFN26A/mpGL4.35[Nluc] Sensor**	
	**Reporter Gene**	Rluc	Fluc	Fluc	Nluc
	**Vector**	pBIND	pGL4.35	mpFN26A	mpGL4.35
**DMSO**		291 ± 47	4 789 ± 1 053	12 116 ± 988	37 457 ± 3 793
**Rosiglitazone**	10 pM	336 ± 54	5 358 ± 1 028	12 251 ± 491	34 729 ± 2 391
	100 pM	361 ± 47	5 306 ± 680	12 799 ± 1 272	39 509 ± 2 453
	1 nM	323 ± 70	5 803 ± 887	15 001 ± 1 153	50 491 ± 4 113
	10 nM	274 ± 29	9 883 ± 2 015	14 934 ± 1 961	122 416 ± 15 659
	100 nM	176 ± 30	13 428 ± 2 516	8 680 ± 1 023	193 978 ± 36 234
	1 μM	136 ± 26	20 583 ± 4 400	6 741 ± 542	235 818 ± 49 563
	10 μM	102 ± 15	26 724 ± 5 559	6 567 ± 566	254 333 ± 40 749

Table S4. Fold induction values observed with pBIND/pGL4.35[Fluc] and mpFN26A/mpGL4.35[Nluc] sensor systems, in uniplex and triplex modes, in cells exposed to 10 μM of each PPAR reference agonist, GW7647 (PPARα), GW501516 (PPARβ) or rosiglitazone (PPARγ).

**Sensor system**	**Mode**	**GW7647**	**GW501516**	**Rosiglitazone**
mpFN26A/ mpGL4.35[Nluc]	Uniplex	13.66 ± 7.13	13.80 ± 4.0	6.67 ± 1.05
	Triplex	19.18 ± 6.14	11.42 ± 1.49	13.14 ± 1.43
pBIND/ pGL4.35[Fluc]	Uniplex	7.02 ± 1.82	33.90 ± 2.57	15.30 ± 3.88
	Triplex	5.32 ± 0.54	6.12 ± 0.72	8.88 ± 1.13

Table S5: **Z-factor values determined for the eleven 96-well plates of the primary screening.** Analysis of 768 cyanobacteria fractions, in twelve plates using the mpFN26A/mpGL4.35[Nluc] sensor system in triplex mode. Positive controls of 10 μM WY14643 (PPARα agonist), 10 μM GW501516 (PPAR β agonist) and 10 μM rosiglitazone (PPARγ agonist) were evaluated in every plate to perform quality control analysis. Z-score values that did not pass the quality control are highlighted in bold. Plate #11 did not pass quality control with any of the agonists tested and it was discharged.

Plate	WY14643	GW501516	Rosiglitazone	Number of hits
#1	**-0.57**	0.41	0.55	14
#2	**-0.21**	0.62	0.69	8
#4	**-3.70**	0.81	0.81	6
**#5**	0.41	0.88	0.87	2
#6	**-1.26**	0.61	0.61	8
**#7**	0.48	0.98	0.87	3
#8	**-0.02**	0.40	0.71	4
#9	**-1.84**	0.13	0.22	2
#10	0.05	0.87	0.93	5
#11	**-0.48**	**-0.16**	**-0.20**	*7*
#12	**-0.88**	0.05	0.45	8

Table S6: Heat map showing the transactivation activity observed in cells upon exposure to fractions of various cyanobacteria strains in the primary screening. Only the 35 strains that showed activity in at least one fraction are represented. Activity (fold induction) was determined with mpFN26A/mpGL4.35[Nluc] sensor system in triplex mode. Hits correspond to either induction (fold induction > 2) or repression (fold induction < 0.5) of PPARs' activity, and their values are presented in numerals. The heatmap has a continuous color scale, with green (maximum fold induction = 9) indicating PPARs induction, and red PPARs repression (minimum fold induction = 0).

		A	B	C	D	E	F	G	H
Nostocales	LEGE 00248	0.439							
	LEGE 00249					2.208			
	LEGE 02266			3.504	5.902	7.127			
	LEGE 06100		2.840						
	LEGE 06105					2.409			2.289
	LEGE 06122*					2.059			
	LEGE 07177*	0.207			5.810	2.807			
	LEGE 07189				2.051				
	LEGE 07189*					2.090			
	LEGE 08334			2.057					
	LEGE 12449		0.464					2.572	
	LEGE 12450							2.707	
	PCC 7107		2.210	3.306	2.737				2.257
Pleurocapsales	LEGE 07179								0.500
Chroococales	LEGE 03274	0.385							
	LEGE 09399						2.257		
	LEGE 91094	0.395				2.069			0.498
Oscillatoriales	LEGE 06078						2.628		
	LEGE 06188						2.750	2.579	
	LEGE 06204		0.447						
	LEGE 07167			2.703	3.304				
Synechococcales	LEGE 03283								2.840
	LEGE 06005			2.037	2.417	2.759			
	LEGE 06013	2.255							
	LEGE 06098			0.462		2.229			
	LEGE 06102	0.325							
	LEGE 06115	0.478		2.190					
	LEGE 06139		4.625	2.159					
	LEGE 06141	0.319			0.424			0.221	
	LEGE 07085			5.575	4.118	2.248			
	LEGE 07171							0.454	
	LEGE 08333							8.746	
	LEGE 10387		0.476						
	LEGE 13457	0.282							
	LEGE 13458	0.413							
	LEGE 15481					2.142			
n.d.	LEGE 00064		2.198						
	LEGE 07227				2.060				
